# Improvement of Physical Activity by a Kiosk-based Electronic Screening and Brief Intervention in Routine Primary Health Care: Patient-Initiated Versus Staff-Referred

**DOI:** 10.2196/jmir.1745

**Published:** 2011-11-22

**Authors:** Matti Leijon, Daniel Arvidsson, Per Nilsen, Diana Stark Ekman, Siw Carlfjord, Agneta Andersson, Anne Lie Johansson, Preben Bendtsen

**Affiliations:** ^1^Center for Primary Health Care ResearchLund University/Region SkåneMalmöSweden; ^2^Department of Medical and Health SciencesDivision of Community MedicineLinköping UniversityLinköpingSweden; ^3^Department of Nursing, Health and CultureUniversity WestTrollhättanSweden; ^4^County Council of ÖstergötlandR&D Department of Local Health CareLinköping UniversityLinköpingSweden; ^5^Motala HospitalDepartment of MedicineMotalaSweden

**Keywords:** Computer-tailored, eHealth, lifestyle behavior, exercise, automated

## Abstract

**Background:**

Interactive behavior change technology (eg, computer programs, Internet websites, and mobile phones) may facilitate the implementation of lifestyle behavior interventions in routine primary health care. Effective, fully automated solutions not involving primary health care staff may offer low-cost support for behavior change.

**Objectives:**

We explored the effectiveness of an electronic screening and brief intervention (e-SBI) deployed through a stand-alone information kiosk for promoting physical activity among sedentary patients in routine primary health care. We further tested whether its effectiveness differed between patients performing the e-SBI on their own initiative and those referred to it by primary health care staff.

**Methods:**

The e-SBI screens for the physical activity level, motivation to change, attitudes toward performing the test, and physical characteristics and provides tailored feedback supporting behavior change. A total of 7863 patients performed the e-SBI from 2007 through 2009 in routine primary health care in Östergötland County, Sweden. Of these, 2509 were considered not sufficiently physically active, and 311 of these 2509 patients agreed to participate in an optional 3-month follow-up. These 311 patients were included in the analysis and were further divided into two groups based on whether the e-SBI was performed on the patient´s own initiative (informed by posters in the waiting room) or if the patient was referred to it by staff. A physical activity score representing the number of days being physically active was compared between baseline e-SBI and the 3-month follow-up. Based on physical activity recommendations, a score of 5 was considered the cutoff for being sufficiently physically active.

**Results:**

In all, 137 of 311 patients (44%) were sufficiently physically active at the 3-month follow-up. The proportion becoming sufficiently physically active was 16/55 (29%), 40/101 (40%), and 81/155 (52%) for patients with a physical activity score at baseline of 0, 1 to 2, and 3 to 4, respectively. The patient-initiated group and staff-referred group had similar mean physical activity scores at baseline (2.1, 95% confidence interval [CI] 1.8-2.3, versus 2.3, 95% CI 2.1-2.5) and at follow-up, (4.1, 95% CI 3.4-4.7, vs 4.2, 95% CI 3.7-4.8).

**Conclusions:**

Among the sedentary patients in primary health care who participated in the follow-up, the e-SBI appeared effective at promoting short-term improvement of physical activity for about half of them. The results were similar when the e-SBI was patient-initiated or staff-referred. The e-SBI may be a low-cost complement to lifestyle behavior interventions in routine primary health care and could work as a stand-alone technique not requiring the involvment of primary health care staff.

## Introduction 

Physical inactivity is acknowledged to be the fourth leading risk factor for global mortality [1]. In Sweden, it has been estimated that physical inactivity contributes to 3.5% of the burden of disease [2]. Hence, effective intervention methods are needed to promote a physically active lifestyle in the population.

Primary health care has been acknowledged as a strategic setting for lifestyle behavior interventions, as indicated by the rapid increase in the number of studies in this field during the last decade [3-6]. There is evidence of both short-term [3-5] and long-term [6] increases in physical activity following counseling provided in primary health care. However, most of these studies were not performed as part of routine care, and they often involved additional personnel and/or patient contacts to those that are usually available. Several barriers to the implementation of lifestyle behavior interventions in routine care have been discussed in the literature, such as insufficient time, high costs, lack of financial reimbursement, perceptions of poor patient adherence to the interventions, limited confidence in providing counseling on lifestyle behaviors, insufficient knowledge about the benefits of physical activity, and lack of appropriate tools to assess and prescribe physical activity [7].

In the light of these implementation challenges, researchers have suggested that the use of interactive behavior change technology (eg, computer programs, Internet websites, and mobile phones) could facilitate the implementation of lifestyle behaviors interventions in primary health care [3,8]. Such technology may address at least some of the barriers to offering face-to-face lifestyle behavior interventions, such as high intervention costs, lack of time, and lack of knowledge. Although there are numerous studies investigating the effectiveness of computer-based and Web-based interventions, they are rarely performed as part of routine care [9,10]. 

Acceptability of computer-based interventions has been reported to be high among patients in primary health care [11,12] and to be highest among those who were referred by staff to perform the intervention [11] and whose doctor examined the results [12]. Hence, implementation of computer-based interventions as an integrated part of patient counseling may facilitate the use of such interventions in lifestyle behavior change. On the other hand, involvement of primary health care staff in computer-based interventions increases intervention costs, which is important because one of the concepts behind these techniques is to provide automated, stand-alone support to lifestyle behavior change at a low cost. Hence, we are interested in whether computer-based interventions are effective as stand-alone intervention methods in routine primary health care. 

An electronic screening and brief intervention (e-SBI) system has been developed by a research team at Linköping University, Sweden. The system consists of a screening questionnaire collecting lifestyle data and an immediate feedback system that reports patient risk level and provides tailored advice for lifestyle behavior change. The e-SBI can be set up to be performed as part of ordinary patient counseling in primary health care or as stand-alone computer stations with touch screens without staff referral. Since it was started in the fall of 2006, the e-SBI has been successively implemented in primary health care in Östergötland County, Sweden. Results describing different aspects of the implementation phase have been reported previously [11,13-15]. We have since begun to evaluate the effectiviness of the e-SBI. We started by focusing on differences between patient-initiated and staff-referred e-SBIs. The initial results of this evaluation for behavior change concerning alcohol intake have recently been published [16]. They showed that the e-SBI had a positive influence on alcohol consumption that did not differ according to whether it was patient-initiated or staff-referred. In the present study, we explored the effectiveness of the physical activity module of the e-SBI and whether it differed between patients who performed the e-SBI on their own initiative and those who were referred to it by primary health care staff.

## Methods

### Study Location and Patients

The study was conducted in Östergötland County, Sweden, which had approximately 420,000 inhabitants during the study period (2007-2009). There were 42 operating primary health care units within the county when the study was performed. The units differed with regard to number of listed patients aged 18 years and over (average 9500, range 4200 to 16,500) and the number of general practitioners (GPs), nurses, and other staff members employed.

The number of primary health care units offering patients the e-SBI was successively extended during the study period from 10 units in 2007 to 28 units in 2009. The included units were situated in both urban and rural areas. The e-SBI was performed anonymously as part of routine health care. Patients performing the e-SBI during a two-year period, from September 2007 to August 2009 and who were not considered to be sufficiently physically active according to the results of the physical activity screening (see the physical activity section below) were included in the study. The patients were further divided into two groups. The first group consisted of patients who performed the e-SBI on their own initiative, hereafter referred to as the *patient-initiated* group. In the second group, hereafter referred to as the *staff-referred* group, the patients were invited to perform the e-SBI after their appointments with primary health care staff. Referrals were made by GPs, nurses, physiotherapists, or other staff members responsible for consultations involving lifestyle behaviors. Each primary health care unit was allowed to decide who should make the referrals.

### The Electronic Screening and Brief Intervention Concept

**Figure 1:**
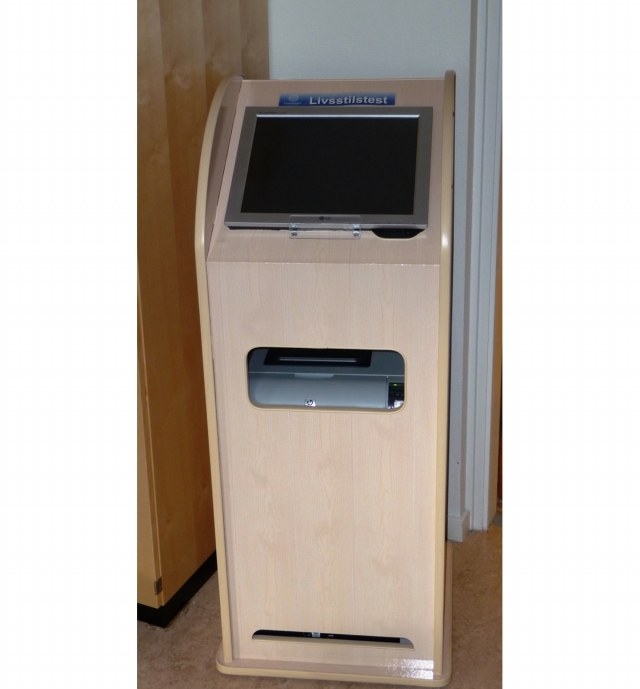
The e-SBI touch screen IT kiosk.

Primary health care units participating in the study were provided with one or two sets of computers, monitors, and printers depending on enrolled patient population size; all were included in stand-alone, touch-screen information technology (IT) kiosks (Figure 1). The same equipment was used for both patient-initiated and staff-referred tests. It was placed in or close to a waiting room in which a poster providing information about the test was displayed. The e-SBI concept was based on previous findings of using e-SBI in student health care and emergency department settings [17-21]. The e-SBI included health-related questions regarding alcohol consumption, physical activity, motivation to change, and attitudes toward performing the test. A personalized written feedback was received, including summaries on the current physical activity level, and printed out at the kiosk after patients completed the tests. In the present study, only physical activity-related data are presented. A question was included in the e-SBI concerning whether the patient was referred to the test by staff or performed it on his/her own initiative.

### Physical Activity

The physical activity measure in the e-SBI concept included two separate questions based on the American College of Sports Medicine/American Heart Association recommendation from 2007 [22]. This recommendations says that adults should reach 5 days of 30 minutes of moderate activity, 3 days of 20 minutes of vigorous activity, or a combination of both (eg, walking briskly for 30 minutes on 2 days during a week and jogging for 20 minutes on 2 other days). Hence, the first question in the e-SBI asked the participants the number of days in a usual week they performed at least 30 minutes (in bouts of at least 10 minutes each) of moderate physical activity, and the second asked the participants the number of days in a usual week they performed at least 20 minutes of vigorous physical activity. A physical activity score was calculated by summing the number of days during which the required amount of moderate or vigorous physical activity was performed. On occasions of combinations of days of moderate and vigorous physical activity, the number of days of moderate physical activity was weighted by 1 and vigorous physical activity by 1.7 (5 days/3 days = 1.7) when calculating the physical activity score. The physical activity score could have a value between 0 and 18.9, and the cutoff for sufficient physical activity set to 5 (fulfilling the American College of Sports Medicine/American Heart Association recommendation).

### Follow-up at 3 Months

After completing an e-SBI but before receiving a personalized printout, each patient was invited to participate in an optional follow-up mail survey 3 months later. Those who accepted this invitation were asked to register their national identification number at the end of the test, and they received a questionnaire by mail 3 months later. Mail addresses were retrieved from the Swedish population register. The mailed questionnaire included the same questions about moderate and vigorous physical activity as used at baseline. A reminder was sent 2 weeks after the follow-up questionnaire to those who had not returned the questionnaire.

Based on their response to the invitation to participate in the follow-up mail survey and completion of the follow-up questionnaire, patients were further categorized into three groups: *nonparticipants* completed the e-SBI but did not agree to participate in the follow-up survey, *no* *n* *responders* completed the e-SBI and agreed to participate in the follow-up survey but did not respond to the follow-up questionnaire, and *responders* provided information at both baseline and follow-up.

### Ethics

Since the data collection was performed as part of routine health care and the data consisted only of responses to a written questionnaire provided by patients who had given informed consent, there was, according to Swedish law, no need for formal ethical approval at the time at which the data collection was started. However, since then―in June 2008―the regulations were changed due to uncertainty about how to distinguish between routine and research data collection. For new studies involving similar data collection methods, ethical approval would now be required.

### Statistics

Baseline data from nonparticipants, nonresponders, and responders were compared to determine the representativeness of participants in the follow-up (responders). Pearson’s chi-square test was used to analyze differences in terms of sociodemographic characteristics. Also, mean (95% confidence interval [CI]) and median (interquartile range [IQR]) physical activity scores were compared (Tables 1). 

Among responders, physical activity score and physical activity score category at baseline and 3-month follow-up were compared between patients who performed the e-SBI on their own initiative and those who were referred to it by primary health care staff. Created were four physical activity score categories: 0, 1 to 2, 3 to 4, and greater than or equal to 5. Pearson’s chi-square test was used to analyze differences in physical activity score category at baseline and at 3-month follow-up, together with comparison of mean (95% CI) and median (IQR) physical activity scores (Table 3). Improvement in physical activity by the e-SBI was assessed from the physical activity score and physical activity score category at follow-up compared with baseline. All statistical analyses were performed using SPSS 18.0 (SPSS Inc, Chicago, IL, USA).

## Results

### Participation

**Figure 2:**
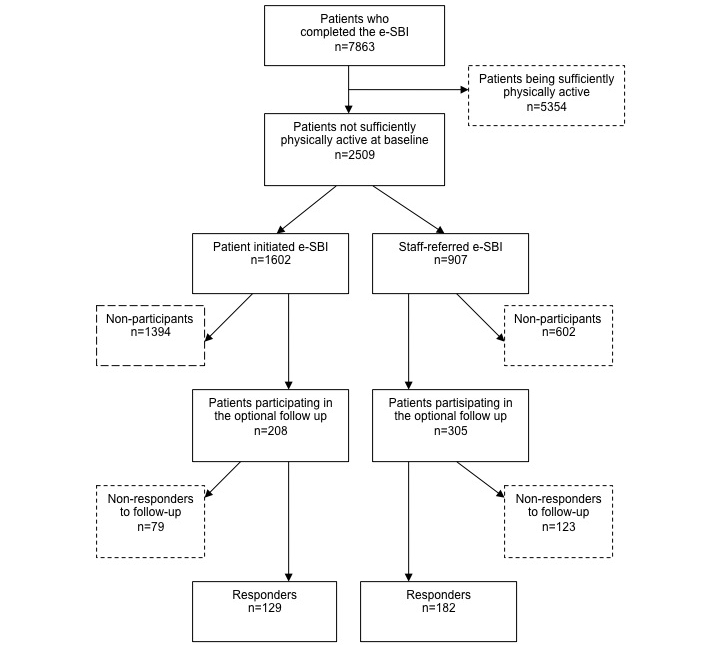
Flowchart of the recruitment of patients.

A total of 7863 patients completed the e-SBI during the two-year sampling period (Figure 2). Of these, 2509 were categorized as not being sufficiently physically active (physical activity score < 5) and were included in the study. Among the included patients, more performed the e-SBIs on their own initiative (1602/2509 or 64%) than were referred to it by primary health care staff (907/2509 or 36%). However, the proportion of patients agreeing to participate in the follow-up was larger in the staff-referred group than in the patient-initiated group at 34% (305/907) versus 13% (208/1602). The final proportion of patients who completed the follow-up (responders) was 20% and 8% in the staff-referred and patient-initiated groups, respectively.

In the patient-initiated group, the proportion of older patients at baseline was significantly higher among responders compared with nonresponders and nonparticipants. However, there were no significant differences in gender distribution or physical activity score among the three groups (Table 1).

In the staff-referred group, the proportion of men was significantly higher among nonresponders compared with the other groups (Table 2). Also, the proportion of older patients was significantly higher among responders compared with nonparticipants. There was, however, no difference in physical activity score among the three groups.

**Table 1:** Patient-initiated e-SBI: baseline characteristics of nonparticipants, nonresponders, and responders

		Nonparticipants	*P* Value, Nonparticipants vs Nonresponders	Nonresponders	*P* Value, Nonresponders vs Responders	Responders	*P* Value, Nonparticipants vs Responders
**Gender, n (%) (*P* = .09)**							
	Men	716 (51)		35 (44)		55 (43)	
	Women	678 (49)		44 (56)		74 (57)	
	Total	1394 (100)	.25	79 (100)	.89	129 (100)	.07
**Age, n (%) (*P* < .001)**							
	18-20	125 (9)		10 (13)		3 (2)	
	21-30	233 (17)		19 (24)		18 (14)	
	31-40	360 (26)		23 (29)		22 (17)	
	41-50	237 (17)		6 (8)		12 (9)	
	51-60	216 (16)		10 (13)		31 (24)	
	≥ 61	211 (15)		11 (14)		42 (33)	
	Total	1382 (100)	.15	79 (100)	< .001	128 (100)	< .001
**Physical activity score**							
	Mean (95% CI)	1.9 (1.8-2.0)		1.8 (1.5-2.2)		2.1 (1.8-2.3)	
	Median (IQR)	2 (0-3)		2 (0-3)		2 (1-3)	

**Table 2:** Staff-referred e-SBI: baseline characteristics of nonparticipants, nonresponders and responders

		Nonparticipants	*P* Value, Nonparticipants vs Nonresponders	Nonresponders	*P* Value, Nonresponders vs Responders	Responders	*P* Value, Nonparticipants vs Responders
**Gender, n (%) (*P* = .06)**							
	Men	319 (53)		78 (63)		92 (51)	
	Women	283 (47)		45 (37)		90 (50)	
	Total	602 (100)	.04	123 (100)	.03	182 (100)	.61
**Age, n (%)(*P*= .001)**							
	18-20	54 (9)		4 (3)		5 (3)	
	21-30	72 (12)		12 (10)		11 (6)	
	31-40	82 (14)		16 (13)		21 (12)	
	41-50	108 (18)		27 (22)		26 (14)	
	51-60	123 (21)		35 (29)		56 (31)	
	≥ 61	156 (26)		28 (23)		63 (35)	
	Total	595 (100)	.11	122 (100)	.18	182 (100)	< .001
**Physical activity score**							
	Mean (95% CI)	2.0 (1.9-2.2)		2.1 (1.8-2.3)		2.3 (2.1-2.5)	
	Median (IQR)	2 (1-3)		2 (1-3)		3 (1-3)	

### Improvement in Physical Activity (Responders Only)

There was no statistically significant difference in physical activity score or physical activity score category between the patient-initiated and staff-referred groups at baseline or at the 3-month follow-up (Table 3). For all responders together, mean physical activity score was significantly higher at 3-month follow-up (4.2, 95% CI 3.8-4.6) compared with baseline (2.2, 95% CI 2.0-2.3). The median (IQR) score increased only slightly, from 2.5 (1-3) to 3 (1-6). However, 44% (136/311) of the patients succeded in becoming sufficiently physical active at the 3-month follow-up.

**Table 3:** Physical activity score and category distribution at baseline and follow-up

Physical Activity Score^a^			Patient-Initiated n = 129	Staff-Referred n = 182	All Responders n = 311
**Baseline**					
	Mean (95% CI)		2.1 (1.8-2.3)	2.3 (2.1-2.5)	2.2 (2.0-2.3)
	Median (IQR)		2 (1-3)	3 (1-3)	2.5 (1-3)
	**Physical activity score category, n (%)^b^**				
		0	24 (19)	31 (17)	55 (18)
		1-2	49 (38)	52 (29)	101 (32)
		3-4	56 (43)	99 (54)	155 (50)
		≥ 5	0 (0)	0 (0)	0 (0)
**3 month follow-up**					
	Mean (95% CI)		4.1 (3.4-4.7)	4.2 (3.7-4.8)	4.2 (3.8-4.6)
	Median (IQR)		3 (1-6)	3 (2-6)	3 (1-6)
	**Physical activity score category, n (%)^c^**				
		0	20 (16)	21 (12)	41 (13)
		1-2	26 (20)	37 (20)	63 (20)
		3-4	31 (24)	40 (22)	71 (23)
		≥ 5	52 (40)	84 (46)	136 (44)

^a^The physical activity score ranged between 0 and 18.9, and the cutoff for being sufficiently physically active was 5. No patients were categorized as sufficiently physically active at baseline according to inclusion criteria.

^b^χ^2^_2_ = 3.99 (*P* = .14), patient-initiated versus staff-referred for categories 0, 1-2 and 3-4 at baseline

^c^χ^2^_2_ = 1.63 (*P* = .65), patient-initiated versus staff-referred for categories 0, 1-2, 3-4 and ≥ 5 at follow-up

Table 4 shows descriptive data of the change in physical activity score category from baseline to follow-up according to physical activity score category at baseline. Of patients with a physical activity score of zero at baseline, 29% (16/55) became sufficiently physically active at the 3-month follow-up. The corresponding proportions for those with physical activity scores of 1 to 2 and 3 to 4 at baseline were 40% (40/101) and 52% (81/155), respectively.

**Table 4:** Change in physical activity score category from baseline to 3-month follow-up in all responders (n = 311)

		Physical Activity Score Category at 3-Month Follow-up			
Physical activity score category at baseline	n	0 %	1-2 %	3-4 %	≥5 %
0	55	35	20	16	29
1-2	101	13	26	22	40
3-4	155	6	17	26	52

## Discussion

In this study, previously sedentary patients in primary health care improved their physical activity 3 months after performing an electronic screening and brief intervention (e-SBI). Overall, 44% of the patients became sufficiently physically active and the improvement in physical activity was similar when the e-SBI was patient-initiated or staff-referred.

These results suggest that the e-SBI is an effective method for promoting a short-term increase in physical activity in patients in primary health care. The e-SBI may be employed as a part of routine care, but there are several factors that need to be taken into account for implementation to be effective. These include staff expectations, perceived need for the innovation to be implemented, compatibility with existing routines, and implementation strategy [11,13-15]. The e-SBI may be used as an integrated part of lifestyle behavior counseling, which may promote greater patient acceptability [11,12] and adherence to the intervention. Patients can be referred by their physician, nurse, or physiotherapist to perform the e-SBI during a visit to the primary health care center. Together, they can then examine the results as part of the consultation, providing background information for physical activity referrals. Alternatively, patients could choose to just bring the printed feedback home for their own use or reference.

The e-SBI could also be used as a stand-alone technique for promoting lifestyle behavior change, as it produces similar results without the involvement of primary health care staff. Posters informing the patients about the e-SBI can be placed in the waiting rooms. This would be an attractive, low-cost option for primary health care. Both patient-initiated and staff-referred e-SBIs may represent cost-effective complements to ordinary face-to-face interventions and may provide sufficient support to those patients who show better acceptance of this kind of technique. This may free up time for patients requiring face-to-face interventions. Although the initial costs of implementating the e-SBI might be high, the running costs, including technical support, would be far less than face-to-face counseling. The e-SBI would, therefore, deliver cost savings in the long run. Besides, the implementation costs for face-to-face interventions, including staff training, may also be high.

In the present study, there was a lower attrition rate at follow-up in the staff-referred group compared with the patient-initiated group. The extra attention/support experienced by the staff-referred group may have promoted continued participation in the study. However, this does not mean that all patients in the staff-referred group had sufficient motivation to improve their physical activity on their own. In the patient-initiated group, patients who remained in the study may have been those with higher internal motivation for behavior change. Hence, the extra attention/support provided to the staff-referred group versus the motivational characteristics of the remaining participants in the patient-initiated group may explain similarities in improvement in physical activity between the groups. The e-SBI (patient-initiated or staff-referred) may be adapted to meet the support needs of individual patients.

The results of the present study can be compared with those of our previous study of the effect of physical activity referral in routine primary health care in Östergötland County [23]. A typical physical activity referral was performed as a face-to-face counseling session by a physician, nurse, or physiotherapist and resulted in a physical activity prescription. Physical activity was followed up after 3 months through a telephone interview, a postal questionnaire, or during a normal return visit. Participants were asked to give the number of days of at least 30 minutes of physical activity of at least moderate intensity during a week [23]. In the present study, 44% of the patients were sufficiently physically active at follow-up. The proportions for patients who were physically active on 0, 1 to 2, and 3 to 4 days per week at baseline were 29%, 40%, and 52%, respectively. The corresponding proportions in our previous study were 29% for all patients, and 26%, 25%, and 40% for patients who were physically active on 0, 1 to 2, and 3 to 4 days per week at baseline, respectively [23]. The differences in proportions between the studies may partly be explained by the different questions used, although both question formats aimed to separate those who reached the recommended activity level from those who did not. In the present study, the number of days of moderate and vigorous physical activity was assessed by two separate questions, which may have promoted reporting of a higher number of days of physical activity compared with the previous study, in which only one question was used [23]. Also, in the study by Leijon et al, all patients were included in the follow-up [23]. In the present study, the follow-up was optional, which may have caused selection bias through inclusion of the more motivated patients. Considering these methodological differences, one may conclude that the e-SBI promotes short-term improvement in physical activity, similar to physical activity referrals. A study in which the e-SBI and physical activity referrals are directly compared would provide valuable information concerning their complementary roles in routine primary health care. 

We are not aware of any comparable e-SBI physical activity study. Carroll et al performed a randomized controlled trial of a theory-based, computerized physical activity intervention in primary health care [24]. However, in their study, physical activity and psychosocial mediators were investigated through mailed surveys, and the responses from the participants were entered into a computer program by research staff. A tailored report generated and designed to motivate and support behavioral change was then mailed back to the participants. There was no significant difference in moderate-to-vigorous physical activity between the intervention group (139 minutes/week) and control group (109 minutes/week) after 6 months of follow-up. The authors reasoned that performing multiple surveys may have caused reactivity to the research protocol, thereby enhancing physical activity. It is known that assessment reactivity can influence intervention outcomes. For instance, Maisto et al showed that less frequent and less comprehensive assessment yielded lower assessment reactivity in a study of alcohol use and alcohol-related consequences [25].

In the first phase of evaluating the effectiviness of the e-SBI in promoting improved behavior concerning physical activity (the present study) and alcohol consumption [16], we have compared the results of patient-initiated and staff-referred e-SBI. A control group representing routine care was not included in the study design. Hence, a limitation of these two stuides is that we are not able to draw any conclusions concerning the effect of complementing routine care with e-SBI. In routine primary health care, it is not always feasible to apply a randomized controlled trial design. However, it may be a necessary complement to the results of our studies and may confirm the effectiveness of the e-SBI in promoting behavior change in routine primary health care. It is also necessary to include longer follow-up periods to determine the long-term effect of the e-SBI.

In the present study, a self-report measure of physical activity was used to assess change in physical activity following the intervention, as it is part of the e-SBI and may be the most feasible way of assessing physical activity in routine primary health care. However, self-report methods suffer from reporting bias, consisting of a combination of reactivity, recall bias, and social desirability [26]. The level of physical activity has been reported to be considerably higher when assessed by self-report methods than when assessed objectively using accelerometers [27]. Objective methods are considered to provide a more accurate measure of physical activity. Hence, the improvement in physical activity in the present study may have been overestimated. Including objective measures of physical activity in future studies may improve our ability to determine the effectiveness of the e-SBI.

There was a large attrition rate at follow-up in the present study, although patients participating in the follow-up were fairly representative of all patients who performed the e-SBI, reducing the risk of selection bias that may otherwise have affected the intervention outcome.

In conclusion, an electronic screening and brief intervention (e-SBI) implemented in routine primary health care improved physical activity for about half of the sedentary patients who agreed to participate in the follow-up. Similar results were obtained when the e-SBI was patient-initiated or staff-referred. The e-SBI may be a low-cost complement to lifestyle behavior interventions in routine primary health care and could work as a stand-alone technique not involving primary health care staff. 

## Abbreviations

CI: confidence interval
e-SBI: electronic screening and brief intervention
GP: general practitioner
